# The prevalence and impact of antimicrobial allergies and adverse drug reactions at an Australian tertiary centre

**DOI:** 10.1186/s12879-015-1303-3

**Published:** 2015-12-16

**Authors:** Jason A. Trubiano, Kelly A. Cairns, Jacqui A. Evans, Amally Ding, Tuan Nguyen, Michael J. Dooley, Allen C. Cheng

**Affiliations:** Department of Infectious Diseases, Alfred Health and Monash University, Melbourne, VIC Australia; Department of Infectious Diseases, Austin Health, Melbourne, VIC Australia; Department of Medicine, University of Melbourne, Melbourne, VIC Australia; Department of Pharmacy, Alfred Health, Melbourne, VIC Australia; Monash University, Melbourne, VIC Australia; Pharmacy Department, Alfred Hospital and Centre for Medication Use and Safety, Monash University, Melbourne, VIC Australia; Infection Prevention and Healthcare Epidemiology Unit, Alfred Health and Department of Epidemiology and Preventive Medicine, Monash University, Melbourne, VIC Australia

**Keywords:** Antibiotic allergy, Antimicrobial stewardship, Antibiotic usage, Allergy

## Abstract

**Background:**

The prevalence and impact of antimicrobial “allergy” labels and Adverse Drug Reactions (ADRs) on antibiotic usage and antimicrobial stewardship initiatives is ill defined. We sought to examine the rate of antimicrobial “allergy labels” at our tertiary referral centre and impacts on antimicrobial usage and appropriateness.

**Methods:**

Two inpatient antimicrobial prevalence surveys were conducted over a 1-week period in November 2013 and 2014 as part of the prospective National Antimicrobial Prescribing Survey (NAPS). Post survey, patients recorded in the NAPS database were assigned to two groups based upon recorded antimicrobial “allergy label” and ADR: (i) Antimicrobial Allergy/ADR (AA) or (ii) No Antimicrobial Allergy/ADR (NAA). Antimicrobial usage and antimicrobial appropriateness were compared between AA and NAA groups.

**Results:**

From 509 identified patients the prevalence of an antimicrobial allergy or ADR was 25 %. The prevalence of “allergy labels”/ADR was 10 % (51/509) for penicillin V/G, 5 % (24/509) cephalosporins, 4 % (22/509) trimethroprim-sulfamethoxazole and 3 % (17/509) aminopenicillins. One thousand and seventy antimicrobials were prescribed during the study periods, the median antimicrobial duration was longer in the AA versus NAA group (6 days vs. 4 days; *p* = 0.018), and proportion of inappropriate antimicrobial prescribing higher in the AA group compared with NAA (29 %; 35/120 vs. 23 %; 86/367, *p* = 0.22). Oral antimicrobial administration was higher in the NAA than AA group (60 %; 177/297 vs. 46 %; 356/793, *p* = 0.0001). The proportion of patients that received a β-lactam was lower in the AA versus NAA group (60 % vs. 79 %, *p* = 0.0001).

**Conclusions:**

In an Australian tertiary referral centre an antimicrobial “allergy” or ADR label was found to significantly impacted on rate of oral antimicrobial administration, beta-lactam usage, antimicrobial duration and antimicrobial appropriateness.

## Background

Antimicrobial “allergy labels” describing Adverse Drug Reactions (ADRs) are frequently reported. Traditionally, antimicrobials have been known to elicit both Type A ADRs (non-immune mediated) and Type B ADRs (immune mediated), the later typically avoided due to fear of phenotypic recurrence [1, 2]. However the prevalence and impact of antimicrobial Type A and B ADRs is not well defined. Despite the varied pathogenesis of antimicrobial ADRs, the approach to antimicrobial allergy labeling is often the same [3, 4]. The impact antimicrobial ADRs have on antimicrobial stewardship, prescribing, infectious diseases practice and restricted antimicrobial usage is relatively unknown [5]. Utilizing an Australian National Antimicrobial Prescribing Survey (NAPS) prospective database we estimate the prevalence of antimicrobial “allergy labels” and ADRs in an Australian inpatient tertiary hospital population receiving antimicrobial therapy. We describe the commonly implicated antimicrobials in ADRs and allergy descriptions. Furthermore, we assess the impact of antimicrobial “allergy labels” and ADRs on antimicrobial appropriateness and restricted antimicrobial usage.

## Methods

The study was performed at The Alfred hospital, a 350-bed tertiary referral center. Two prevalence surveys were conducted over a 1 week period in November 2013 and 2014 as part of the prospective National Antimicrobial Prescribing Survey (NAPS) [6]. The NAPS was developed in 2011 to assess Australian health care facility antimicrobial prescribing practices. The NAPS identifies inpatients receiving antimicrobial therapy during the prevalence periods, prospectively recording patient antimicrobial usage (agent, dose, frequency, route, duration) and baseline demographics (age, sex, admitting unit, allergy history), infection history (infective diagnosis) and antimicrobial appropriateness score. The NAPs captures recorded patient antimicrobial “allergies” and ADRs from medical records and/or drug charts.

After NAPS data collection patients were assigned to one of two groups based upon the presence of a recorded antimicrobial “allergy label” or ADR: (i) Antimicrobial Allergy/ADR (AA) or (ii) No Antimicrobial Allergy/ADR (NAA). Study investigators JAT and JAE independently defined patient AA description(s) as per published definitions as either likely (i) Type A, (ii) Type B (Class I – IV), or (iii) Unspecified (U) [7].. When consensus could not be reached a third study investigator was recruited for a tie-breaking vote. Antimicrobial usage and antimicrobial appropriateness were compared between AA and NAA groups.

An infectious diseases physician defined antimicrobial appropriateness according to NAPS criteria: 1 (optimal), 2 (adequate), 3 (suboptimal), 4 (inadequate) or 5 (not assessable) [6]. A score of one or two was considered to be ‘appropriate’ and three or four as ‘inappropriate’.

A Type A ADR was defined as a non-immune mediated reaction that relates to the predictable pharmacological properties of a drug. A Type B ADR was defined as an immune mediated reaction that does not related to the predictable pharmacological properties of a drug [1]. This was subcategorized into one of four classes: (i) Immediate (IgE), (ii) accelerated or delayed (Cytotoxic IgG), (iii) accelerated or delayed (Immune complex IgG) and (iv) Delayed (T cell-mediated) [2, 7]. The duration of antibiotic therapy was the number of days from the recorded commencement of antibiotic therapy until the date of the survey. For this study, restricted broad-spectrum antibiotic was defined as a carbapenem, fluoroquinolone or glycopeptide antibiotic. ‘De-labeling’ is defined as the removal or revision of an antibiotic allergy label following clinical allergy assessment and/or allergy testing or re-challenge [5]. Alfred Health Human Research Ethics Committee approval was obtained for NAPS data collection.

### Statistical analysis

Categorical variables were summarized using frequency and percentage and compared between groups using a chi-square test. Continuous variables were summarized using mean and standard deviation (SD) or median and inter-quartile range as appropriate and compared using a paired t-test or Wilcoxon signed-rank test as appropriate. A univariate negative binomial regression model was utilized, a variant of a poisson regression allowing for over-dispersion, to test the null hypothesis of no difference in cumulative days of antimicrobial exposure between groups. Standard errors were estimated using the robust Huber White sandwich estimator.

## Results

We identified 509 patients according to NAPS inclusion criteria from the combined point prevalence periods, 245 from 2013 and 264 from 2014. The baseline cohort demographics are outlined in Table 1. Twenty-five percent (128/509) of patients identified in the NAPs database had a documented report of “antimicrobial allergy” or ADR (AA). The prevalence of “allergy labels”/ADR was 10 % (51/509) for penicillin V/G, 5 % (24/509) cephalosporins, 4 % (22/509) trimethroprim-sulfamethoxazole/sulphonamide antibiotics and 3 % (17/509) aminopenicillins. The distribution of patients between admitting units was similar in the AA and NAA groups (*p* = 0.58), however a higher male predominance in the NAA versus AA group was noted (66 % vs. 49 %, *p* = 0.007).

**Table 1:** Baseline demographics for NAPS cohort (2013-2014)

Cohort demographics	AA	NAA	Total
	*N* = 128	*N* = 381	*N* = 509
Age (years)			
Median	58	59	59
Sex			
Male	63 (49)	252 (66)	315 (61)
Admitting Unit			
Specialist medical	73 (57)	160 (42)	233 (46)
General medical	23 (18)	40 (11)	63 (12)
General surgical	6 (5)	31 (8)	37 (7)
Specialty surgical	25 (2)	147 (39)	172 (34)
Other^a^	1 (1)	3 (1)	4 (1)
Immunosuppressed^b^			
Yes	33 (26)	76 (20)	109 (21)
Total drug allergies	128 (100)	5 (1)	133 (26)
Total antimicrobials administered	297	773	1070
Route of antimicrobial administration (*n* = 1070)			
Oral	177(60)	356 (46)	533 (50)
Intravenous	102 (34)	371 (48)	473 (44)
Other^c^	18 (6)	46 (6)	64 (6)

^a^Emergency (3), Intensive care unit (1)

^b^Haematology, oncology, lung and heart transplant units

^c^Inhaled, topical

### Antimicrobial allergy descriptions

In the 128 patients reporting one or more allergies/ADRs, there were 202 antimicrobial allergies and ADRs recorded in the allergy record for the 128 patients. Of these 202 antimicrobials, 102 (50 %) were considered likely Type B, 48 (24 %) Type A, 51 (25 %) unspecified and 1 % anaphylactoid (2/202). Of those Type B ADRs, 39 % (40/102) had clinical findings consistent with Class I (immediate) reactions and 61 % (62/102) Class IV (delayed). Fifty-four percent (109/202) of reported antimicrobial ADRs were toward a β-lactam, 62 % (67/109) Type B, 17 % (19/109) Type A and 21 % (23/109) unspecified. The complete list of antimicrobial allergies and reported ADRs are demonstrated in Table 2.

**Table 2:** Reported antimicrobial allergies and ADRs for the AA cohort

Reported Antimicrobial Allergy or ADR	Total
	*N* = 202 (%)
*Beta*-*lactams*	109 (54)
Penicillin^a^	54
Amoxicillin/Ampicillin	12
Amoxicillin-clavulanate	5
Flucloxacillin	3
Ticarcillin-clavulanate	5
Piperacillin-tazobactam	4
Cephalosporins (not specified)	3
Cephalexin/Cephazolin	14
Cefuroxime	2
Ceftriaxone	1
Ceftazadime	3
Cefepime	1
Meropenem	2
*Non*-*beta lactams*	93 (46)
Vancomycin	7
Clindamycin/lincamycin	6
Trimethoprim-sulfamethoxazole	21
Dapsone	5
Clarithromycin	2
Doxycycline	2
Erythromycin	11
Roxithromycin	4
Metronidazole	2
Tobramycin	4
Aminoglycosides (NS)	1
Norfloxacin	1
Ciprofloxacin	7
Moxifloxacin	3
Antiretroviral therapy	5
Azoles^b^	6
Other^c^	6

^a^Penicillin – Penicillin V, Penicillin G. ^b^Ketoconazole, fluconazole, voriconazole

^c^Quinine (1), Linezolid (1), Amphoterician (1), clotrimazole (1), Rifampacin (1), terbinafine (1)

### Antimicrobial usage and appropriateness

From the 509 patients, 1070 antimicrobials were prescribed during the study periods, 297 for the AA group versus 773 for the NAA group. The top five antimicrobial agents administered for the AA and NAA groups are displayed in Table 3. The median antimicrobial duration was longer in the AA versus NAA group (6 days vs. 4 days; *p* = 0.018), whilst there was a non-significant increase in the proportion with inappropriate antimicrobial prescribing in the AA group compared with NAA (29 %; 35/120 vs. 23 %; 86/367, *p* = 0.22). Oral antimicrobial administration was higher in the NAA than AA group (60 %; 177/297 vs. 46 %; 356/793, *p* = 0.0001). The proportion of patients that received a β-lactam or cephalosporin was lower in the AA versus NAA groups, 60 % vs. 79 % (*p* = 0.0001) and 24 % vs. 43 % (*p* = 0.05) respectively. There was no difference in the proportion of AA versus NAA patients that received a restricted antimicrobial, 25 % vs. 24 % (*p* = 0.44). Sixty three percent (56/89) of patients reported to be allergic to at least one β-lactam still received a β-lactam antibiotic.

**Table 3:** The most frequent antimicrobials used (in order) for patients with and without antimicrobial “allergy labels” (AA vs. NAA)

AA group	
(*n* = 278)	
1	Ciprofloxacin 15 (5 %)
2	Cephazolin 13 (5 %)
3	Ceftazadime 12 (4 %)
4	Ceftriaxone 11 (4 %)
5	Meropenem 10 (4 %)
NAA group	
(*n* = 792)	
1	Cephazolin 69 (9 %)
2	Ceftriaxone 59 (7 %)
3	Piperacillin-tazobactam 47 (6 %)
4	Ciprofloxacin 40 (5 %)
5	Amoxicillin – clavulanate 33 (4 %)

It was estimated that the AA compared to NAA patients had a non-statistically significant 38 % increase in antimicrobial days exposure (R 0.328, 95 % CI-0.72–0.72, *p* = 0.11) and 31 % increase in antibiotic days exposure (R 0.27, 95 % CI:-0.14–0.68, *p* = 0.19).

## Discussion

By utilizing single center data from the NAPS survey, we were able to demonstrate a high prevalence of total antimicrobial “allergy labels” and ADRs (25 %) in our mixed inpatient population receiving antimicrobial therapy, 55 % of which were consistent with a non-immediate reaction. Our findings are broadly consistent with other reported international studies, but with some interesting differences. We found a quarter of patients had been labeled as having an “allergy” which is at the higher end of previously reported studies [5, 8, 9]. Charneski et al. demonstrated in a large group of general medical and surgical patients that 11 % had an antimicrobial “allergy label” [3]. Lee et al. revealed 25 % antibiotic allergy prevalence, however with a higher rate of immediate reactions than illustrated in our cohort (32 % vs. 20 %) [10].

Identifying patients with either known drug side effects (Type A) or non-severe delayed reactions (Type B) may potentially allow for supervised re-challenge to aid appropriate antimicrobial therapy [11]. However, recent reports suggest this definition may require re-evaluation, as Type A reactions may in fact be dose dependent and genetically predisposed, whilst Type B in some cases non-immune mediated (e.g. non-IgE mediated mast cell activation by fluoroquinolones) [1, 12, 13]. Nonetheless, we identified that 54 % of our study population have likely non-immediate ADRs, highlighting a potential for direct re-challenge and “de-labeling” in those unlikely to be genetically predisposed [14]. β-lactams are often the preferred therapy in many invasive infections, yet often avoided due to a distant history of non-immediate allergy or Type A ADR. The lower uptake of β-lactams in our NAA group may be potentially addressed in the future by direct re-challenge or formal SPT/IDT programs[14]. Clinical programs that have utilized antibiotic re-challenge in ‘low risk’ phenotypes, predominately ‘unknown’ or Type A ADRs, have demonstrated an increase in β-lactam usage post implementation [15]. Interestingly, 60 % of our patients with a β-lactam “allergy” received a β-lactam; the resultant proportion that was subsequently “de-labeled” is unclear. The importance of “de-labeling” strategies is strengthened by studies that have highlighted that an antibiotic “allergy label” is associated with increased costs, antibiotic exposure and resistant organism generation [4, 5, 16].

This study is limited by small study numbers, recall bias of true ADR descriptions by patients and assessment of prescribing over short study periods. Whilst clarification of which patients had “mislabeled” AA would be preferred, the fact that most clinicians are known to act upon these labels without clarification is significant [17]. The study does highlight the need for both a better understanding, as well as a management strategy to assess patients with antimicrobial allergy/ADR “labels”. Although the study had limited power to determine differences in prescribing practices, ciprofloxacin was the most frequently used antimicrobial in the AA group and meropenem was represented in the “top 5”, albeit with no appreciable difference in restricted antimicrobial usage between the groups. Inappropriate prescriptions were more frequently identified in AA patients, and although not statistically significant, this ‘signal’ requires further evaluation in larger cohorts. Furthermore, the AA group had an increased exposure to antimicrobials (i.e. duration), which we intend to also explore more fully in a larger dataset.

## Conclusion

We identified an antimicrobial “allergy label” prevalence of 25 % in our tertiary referral center, 10 % for a penicillin “allergy label”. The majority of adverse drug reactions recorded were against β-lactams (50 %), with a predominance of non-immediate ADRs. The high rate of β-lactam use in patients with a β-lactam “label” raises questions about the validity of these ADRs and/or presence of a selective side chain allergy. A higher amount of intravenous antimicrobial administration and trend toward increased inappropriate antimicrobial prescription in the AA group was also noted. Further assessment of the entire NAPS database, paying particular attention to the highest users of antimicrobial therapy (i.e. immunosuppressed) may further clarify if the differences seen in antimicrobial exposure are significant.

## Abbreviations

ADR: Adverse drug reactions
AA: Antimicrobial allergy
NAA: No antimicrobial allergy
Type A ADR: An adverse drug reaction that is secondary to a known drug side effect and not immune mediated
Type B ADR: An adverse drug reaction that is secondary to an immune mediated mechanism

## References

[1] Han L, Undem BJ, Kulka M, McNeil BD, Pundir P, Meeker S (2015). Identification of a mast-cell-specific receptor crucial for pseudo-allergic drug reactions. Nature.

[2] Gell PG (1963). Clinical aspects of immunology. 2nd ed. Gell PG, Coombs RRA., editor.

[3] Charneski L, Deshpande G, Smith SW (2011). Impact of an antimicrobial allergy label in the medical record on clinical outcomes in hospitalized patients. Pharmacotherapy.

[4] Macy E (2014). Penicillin and beta-lactam allergy: epidemiology and diagnosis. Curr. Allergy Asthma Rep.

[5] Trubiano J, Phillips E (2013). Antimicrobial stewardship’s new weapon? A review of antibiotic allergy and pathways to ‘de-labeling’. Curr Opin Infect Dis.

[6] team Npc. NAPS - National Antimicrobial Prescribing Survey 2015 [cited 2015 1st March 2015]. Available from: http://www.safetyandquality.gov.au/publications/antimicrobial-prescribing-practice-in-australianhospitals-results-of-the-2014-national-antimicrobial-prescribing-survey/. (Accessed 12 December 2015).

[7] Pichler W. Drug Hypersensitivity. Pichler W, editor: S Karger Pub; 2007

[8] Casale TB, Condemi J, Greenberger PA, Shepherd GM, Sogn DD, Evans R (1992). Results of the national institute of allergy and infectious diseases collaborative clinical trial to test the predictive value of skin testing with major and minor penicillin derivatives in hospitalized adults. Archives of internal medicine.

[9] Gadde J, Spence M, Wheeler B, Adkinson NF (1993). Clinical experience with penicillin skin testing in a large inner-city STD clinic. Jama.

[10] Postelnick MJ, Greenberger PA, Peterson LR, Lee CE, Zembower TR, Fotis MA (2000). The incidence of antimicrobial allergies in hospitalized patients: implications regarding prescribing patterns and emerging bacterial resistance. Archives of internal medicine.

[11] Rawlins MD, Thompson JP (1977). Textbook of adverse drug reactions.

[12] Phillips EJ (2015). Classifying ADRs - does dose matter? British journal of clinical pharmacology.

[13] White KD, Chung WH, Hung SI, Mallal S, Phillips EJ (2015). Evolving models of the immunopathogenesis of T cell-mediated drug allergy: the role of host, pathogens, and drug response. J. Allergy Clin. Immunol.

[14] Bourke J, Pavlos R, James I, Phillips E (2015). Improving the effectiveness of penicillin allergy De-labeling. J Allergy Clin Immunol Pract.

[15] Blumenthal KG, Shenoy ES, Varughese CA, Hurwitz S, Hooper DC, Banerji A (2015). Impact of a clinical guideline for prescribing antibiotics to inpatients reporting penicillin or cephalosporin allergy. Ann. Allergy Asthma Immunol.

[16] Macy E, Contreras R (2014). Health care use and serious infection prevalence associated with penicillin “allergy” in hospitalized patients: A cohort study. J. Allergy Clin. Immunol.

[17] Fehily SR, Stuart RL, Horne K, Korman TM, Dendle C (2015). Who really knows their patients’ penicillin adverse drug reaction status? A cross-sectional survey. Intern. Med. J.

